# Completely resected follicular dendritic cell sarcoma of the posterior mediastinum: report of a case

**DOI:** 10.1186/s40792-016-0155-4

**Published:** 2016-03-21

**Authors:** Ryo Miyoshi, Makoto Sonobe, Ei Miyamoto, Hiroshi Date

**Affiliations:** Department of Thoracic Surgery, Kyoto University Hospital, Shogoin-Kawara-cho 54, Sakyo-ku, Kyoto, 606-8507 Japan

**Keywords:** Follicular dendritic cell sarcoma, Posterior mediastinum, 18-fluorodeoxyglucose positron emission tomography, Surgery

## Abstract

Follicular dendritic cell sarcoma is a rare malignant neoplasm originating from follicular dendritic cells, and most of them develop in lymph nodes of the head and neck. One third of follicular dendritic cell sarcomas occur in the extranodal sites such as the tonsils, mesentery, and retroperitoneal organs, but those of mediastinal origin are rare. Here, we present the case of a 16-year-old female with a large follicular dendritic cell sarcoma of posterior mediastinal origin. The tumor was found by a chest X-ray mass examination at her high school, and she had no subjective symptoms or significant past medical history. The tumor was diagnosed as a follicular dendritic cell sarcoma by computed tomography-guided needle biopsy. Although the tumor compressed the mediastinal organs and showed moderate uptake in 18-fluorodeoxyglucose positron emission tomography imaging, it was completely resected through posterolateral incision. Histological examination revealed that spindle-shaped tumor cells formed fascicular or storiform pattern with cellular pleomorphism. By immunohistochemical examination, the tumor cells were found to be positive for CD21 and follicular dendritic cell antigen. Two years after surgery, the patient remains alive with no signs of tumor recurrence.

## Background

Follicular dendritic cell sarcoma (FDCS) is a rare malignant neoplasm originating from follicular dendritic cells, and most FDCSs develop in lymph nodes of the head and neck [1–4]. One third of FDCSs occur in the extranodal sites such as the tonsils, mesentery, and retroperitoneal organs. However, FDCS of mediastinal origin is rare [4]. Here, we report a case of a completely resected FDCS originating from the posterior mediastinum.

## Case presentation

**Fig. 1:**
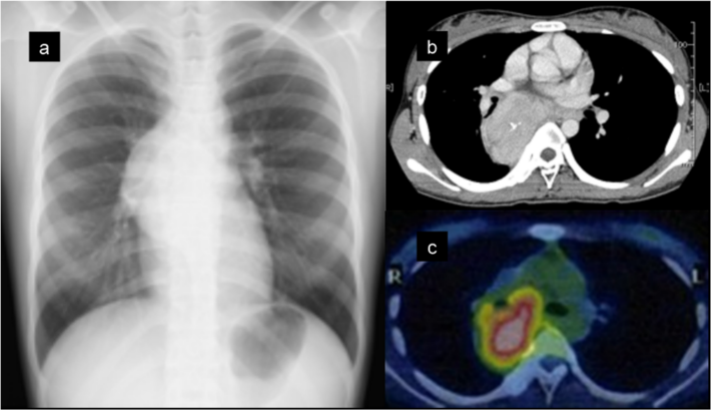
Preoperative imaging studies. Chest X-ray film showed a large para-cardiac mass in the right mediastinum (**a**). Contrast-enhanced computed tomography showed a heterogeneous attenuation within the mass and a focus of coarse calcification (**b**). 18-fluorodeoxyglucose (FDG) positron emission tomography/computed tomography showed moderate FDG uptake within the mass (**c**). *Pink-colored area* indicates that the maximum standardized uptake value was 4.0 or more

A 16-year-old otherwise healthy female visited a local hospital because of detection of a mediastinal tumor at a school screening. Her vital signs were normal, and physical examination was completely unremarkable. The blood test was also normal. Chest X-ray (Fig. 1a) and chest computed tomography (CT) scan (Fig. 1b) showed a large well-defined mass in the right posterior mediastinum. The mass was 8 cm in diameter and compressed the surrounding organs (Fig. 1b). 18-fluorodeoxyglucose positron emission tomography-CT (FDG-PET/CT) showed a moderate uptake with a standardized uptake value of 4.5 in the mass (Fig. 1c). A CT-guided needle biopsy revealed spindle-shaped tumor cells. Immunohistochemically, the tumor cells were positive for CD21 and fascin (data not shown). These results highly suggested FDCS. The patient was referred to our hospital for resection of the tumor because complete resection of localized FDCS was reported to provide better survival [2, 4]. We started surgery via a right posterolateral incision and fifth intercostal thoracotomy. Combined right lower lobectomy of the lung was planned in the event that the tumor had invaded the right lower lobe. The tumor did not infiltrate into the lung or surrounding organs, but highly vascularized adhesion to the right lower lobe and esophagus was observed, which could be dissected. We completely resected the tumor together with the para-esophageal lymph nodes adjacent to the tumor. The operation time was 180 min, and blood loss was 575 mL.

**Fig. 2:**
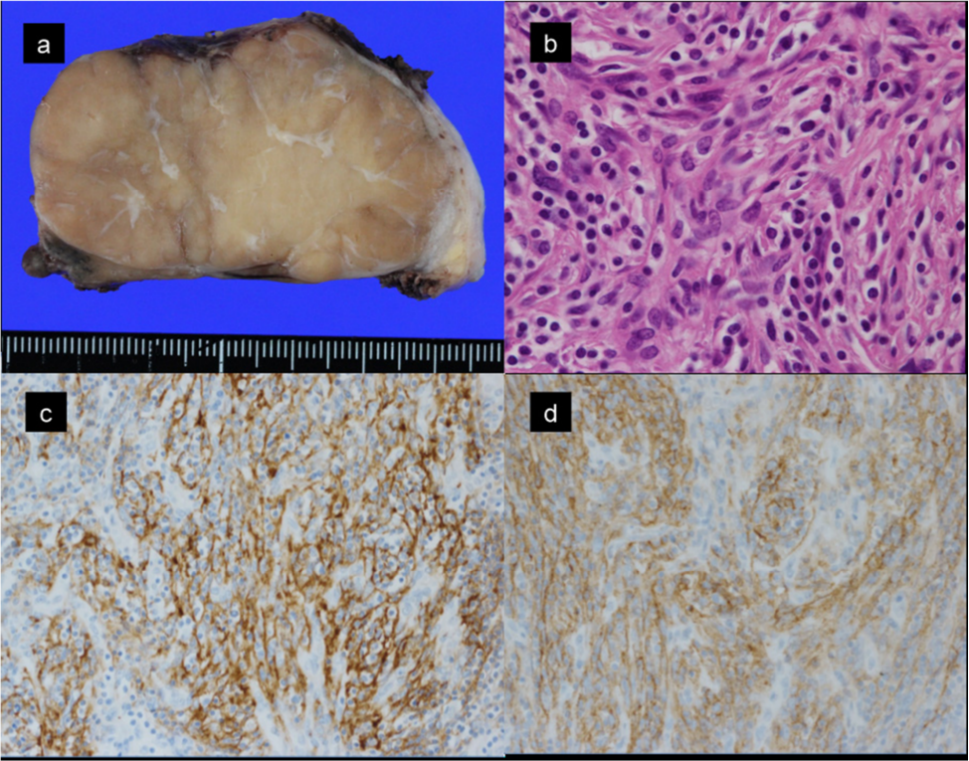
Pathological examinations. The cut surface of the resected tumor had a heterogeneous appearance (**a**). Microscopic examination (hematoxylin-eosin staining, original magnification: ×400) revealed spindle-shaped tumor cells with pleomorphic nuclei and eosinophilic cytoplasm. Infiltration of small lymphocytes was observed around the tumor cells (**b**). Immunohistochemical staining of tumor cells for CD21 (**c**) and FDC antigen (**d**) revealed membranous and cytoplasmic positivity in the tumor cells (original magnification: ×100). The antibody used for FDC antigen was the anti-follicular dendritic cell antibody, Clone CNA.42 (DAKO Japan Co. Ltd, Tokyo)

The tumor was 80 × 65 × 40 mm in size and 110 g in weight. It was well circumscribed, and the cut surface was pale yellow and heterogeneous with fibrous septa (Fig. 2a). Microscopically, spindle-shaped tumor cells were found to form fascicles and storiform pattern. Tumor cells had eosinophilic cytoplasm. Abundant nuclear pleomorphism was observed among the neoplastic cells. Lymphocytic infiltration around the tumor cells was also observed (Fig. 2b). Cellular atypia, necrosis, and mitotic figures were rarely observed. Immunohistochemical analyses revealed that the tumor cells were positive for CD21 (Fig. 2c) and follicular dendritic cell antigen (Fig. 2d), but were negative for CD68, S-100, anaplastic lymphoma kinase, and Epstein-Barr virus-encoded small RNA (data not shown). These findings were compatible with FDCS. There was no evidence of the neoplasm in the dissected para-esophageal lymph nodes.

She had a benign postoperative course and was discharged 8 days after surgery. She received no adjuvant therapy. There has been no evidence of recurrence 2 years after surgery as observed during biannual follow-up with CT.

### Discussion

FDCS is a rare low-grade malignant neoplasm arising from the primary and secondary lymphoid follicles. It was first described in 1986 [1], and specific immunohistochemical markers such as CD21, CD23, CD35, and fascin have been identified. Reactivity to these markers is necessary for definitive diagnosis of FDCS [1–6]. Possible associations with Epstein-Barr virus and Castleman’s disease have also been reported [7]. Recent pooled analysis has shown that FDCS generally occurs in young to middle-aged adults, without apparent sex predilection. FDCS usually presents with painless cervical or intra-abdominal lymphadenopathy, or as a mass in extranodal organs such as the head and neck, liver, lung, spleen, or retroperitoneal organs. Mediastinal origin is uncommon, and only 20 of 334 reported cases arose in the mediastinum [3]. According to the reports of mediastinal FDCS with specification of size, location, and imaging studies [2–18], many of the mediastinal FDCS cases developed in the subcarinal region as in our case [8–10, 12–15]. Usually several lymph nodes reside in the subcarinal region, and these can be a site for development of FDCS. The FDCS in our case most likely originated from a subcarinal lymph node although histological evidence could not be obtained to verify this. Some FDCSs develop in the anterior mediastinum [16–18] or para-tracheal region [11], which are usually approximately 10 cm in diameter and range in size from 2.5 to 13.4 cm [2–18]. Imaging studies by CT show that mediastinal FDCS are usually well defined and with heterogeneous soft tissue attenuation, accompanied by the areas of calcification [8]. FDG-PET/CT shows moderate or high uptake within the tumor, suggesting hypermetabolism of the FDCS [8]. In many reported cases of mediastinal FDCS, complete resection was possible and postoperative radiation therapy was frequently performed [2–11, 13–18]. The prognosis of mediastinal FDCS is unknown because long-term outcome beyond 2 years has not been described in reported cases [2–18]. However, good prognosis has been reported in complete resection cases and complete resection with postoperative irradiation cases as a whole [4] while poor prognosis of non-surgical cases (chemotherapy and / or radiotherapy) has been reported [2, 4, 12]. However, a successful case with multimodality treatment for mediastinal FDCS has been reported [19].

In our case, the FDCS showed an uncommon clinical presentation as a mid- to posterior mediastinal tumor. We could obtain a definitive diagnosis of FDCS by CT-guided needle biopsy in order to facilitate the decision to perform initial operation. Dissection of the large and hypermetabolic FDCSs of mediastinal origin seems to be possible as shown in our case as well as in those reported by Chow et al. [9] and Leipsic et al. [15]. However, Lee et al. reported that right pneumonectomy was needed for complete resection of mediastinal FDCS [10], and Voigt et al. [11] and Ceresoli et al. [12] reported that complete resection of mediastinal FDCS could not be achieved due to direct invasion into the trachea-bronchial tree or esophagus. Therefore, resectability of mediastinal FDCS is difficult to predict from the imaging studies, and the appropriate approach and extent of resection for mediastinal FDCS should be considered during surgery according to the intraoperative findings.

Adjuvant chemotherapy and/or radiotherapy after complete resection are considered for FDCS with poor prognostic features such as extensive necrosis, a mass larger than 6 cm, cytologic atypia, intraabdominal location, and a high proliferative index that suggests a high mitotic activity [3]. However, there is no evidence that adjuvant treatment contributes to better survival [4]. In our case, adjuvant therapy was not performed because the tumor was completely resected without any surrounding lymph node metastasis, the large tumor size (8 cm) was the only poor prognostic factor, and the effectiveness of adjuvant therapy was uncertain. The patient has no clinical or radiological evidence of recurrence and metastasis 2 years after surgery. However, she will require continued monitoring in order to validate the adequacy of our decision to not perform adjuvant therapy.

## Conclusions

We report a case of complete resection of a rare tumor, mediastinal FDCS. Although the evaluation of resectability for mediastinal FDCS is difficult and appropriate management of it is unclear, complete resection and detailed histological evaluation to determine the necessity of adjuvant therapy are preferable.

## Consent

Written informed consent was obtained from the patient for publication of this case report and any accompanying images. A copy of the written consent is available for review by the Editor-in-Chief of this journal.

## Abbreviations

CT: computed tomography
FDCS: follicular dendritic cell sarcoma
FDG-PET: 18-fluorodeoxyglucose positron emission tomography
